# A case of multiple basal cell carcinomas in a patient with MUTYH gene mutation

**DOI:** 10.1016/j.jdcr.2025.02.025

**Published:** 2025-03-11

**Authors:** Christopher Madden, Josephine D'Angelo, Paul Blackcloud

**Affiliations:** a The State University of New York Downstate College of Medicine, Brooklyn, New York; b Department of Dermatology, University of Rochester, Rochester, New York

**Keywords:** basal cell carcinoma, BCC syndromes, DNA repair gene, genetic testing, MUTYH, MUTYH-associated polyposis

## Introduction

Certain germline and somatic mutations have been implicated in the pathogenesis of basal cell carcinoma (BCC). There are several syndromes associated with a predisposition to BCC, which are very rare with a high degree of penetrance.^1^ The most common BCC syndrome, Gorlin syndrome or basal cell nevus syndrome, is an autosomal dominant (AD) disorder most frequently associated with PTCH1 mutation, while PTCH2 and SUFU are less commonly implicated.^2^ The diagnosis of Gorlin syndrome is made by the presence of 2 major or one major and 2 minor clinical criteria. Major criteria include multiple (>2) BCCs or 1 BCC before age 20, odontogenic keratocysts of the jaw, palmar or plantar pitting, bilamellar calcification of the falx cerebri, and bifid or fused ribs.^3^ Minor criteria include, but are not limited to, medulloblastoma, increased head circumference, congenital malformations, skeletal abnormalities, radiologic abnormalities, and cardiac or ovarian fibromas.^3^ Other BCC syndromes include Rombo syndrome (AD), Bazex-Dupré-Christol syndrome (X-linked dominant > AD), Brooke-Spiegler syndrome (AD), multiple hereditary infundibulocystic BCC (AD), Schopf-Schultz-Passarge syndrome (autosomal recessive > AD), and xeroderma pigmentosum (autosomal recessive).^1^ Recent cases of patients with multiple BCCs have revealed mutations in MUTYH without other mutations in known BCC-associated genes.

## Case report

A 61-year-old Caucasian male, Fitzpatrick type II, with a past medical history of anxiety, asthma, and rectal adenocarcinoma status-post transanal endoscopic microsurgery presented to dermatology in 2010 for a suspicious lesion on the right antecubital fossa. Biopsy was performed revealing a nodular BCC. The patient has since been diagnosed with 32 additional BCCs of the nodular, infiltrating, and superficial types at routine follow-up visits. Other relevant dermatologic history includes 3 squamous cell carcinomas and a spitz nevus. Family history was significant for single melanomas in the patient's sister (age 38) and maternal uncle (age 65), multiple BCCs in the patient's father (approximately 6 as per patient), and colon cancer in his maternal uncle (age 65) and maternal cousin (age 40). He has no smoking history but had blistering sunburns when he was younger.

Given his extensive history of BCCs and rectal adenocarcinoma, the patient was referred for genetic testing. Myriad MyRisk genetic testing was performed and revealed a variance of uncertain significance in CDKN2A (p16INK4a) c.-33G>C and MUTYH c.679G>A. Testing for PTCH1 and SUFU was negative, which reduced the likelihood of Gorlin syndrome or other known BCC syndromes. CDKN2A, cyclin-dependent kinase inhibitor 2A, is a major gene associated with melanoma. A variance of uncertain significance implies that there is inadequate evidence in the literature for that variant's pathogenicity, and the c.-33g>c single nucleotide variant of CDKN2A has not previously been implicated in BCC. Given previous reports of patients with multiple BCCs and MUTYH variance, the MUTYH variance of uncertain significance warranted additional review for a possible relationship to this patient's history of rectal adenocarcinoma and extensive BCCs.

## Discussion

The MUTYH gene encodes a DNA glycosylase enzyme, which is a component of the base-excision repair system that is responsible for preventing oxidative damage.^4^^,^^5^ Mutations in this gene have primarily been linked to MUTYH-associated polyposis (MAP), an AD disorder associated with increased risk for colorectal adenomas and carcinomas.^6^ MAP has also been associated with increased relative risk for both melanoma and nonmelanoma skin cancers and a variety of benign skin tumors.^6^ A 2018 study of 61 patients with recurrent BCC found that 19.7% of these patients harbored mutations in DNA repair genes, including MUTYH.^4^ Two recent cases of a 35-year-old female and a 39-year-old male with recurrent BCC also reported mutations in MUTYH.^5^^,^^7^

Our case builds on the growing literature of the link between BCC and pathogenic MUTYH mutations. These cases may suggest a role for early skin cancer screening in patients with known MUTYH mutations. Additionally, these cases may highlight a potential need for gastrointestinal screening in patients presenting with recurrent or multiple BCCs. In situations where patients present with multiple BCCs but do not meet clinical criteria for Gorlin syndrome or other known BCC syndromes, it may be worth considering genetic screening given that MUTYH mutations are also associated with other cancers, including MAP, and if present, these mutations may warrant earlier gastrointestinal screening. While there are established guidelines for screening for MAP from the American College of Gastroenterology and National Comprehensive Cancer Network, there is limited guidance on genetic testing for BCC syndromes.^8^^,^^9^ Furthermore, guidelines from the American College of Medical Genetics and Genomics advise that variants of unknown significance, like in our case, should not be used in clinical decision-making.^10^ Importantly, as more cases are identified and added to the literature, these variants may become significant and ultimately result in the establishment of screening guidelines that impact patient care. We recognize that availability and cost of genetic testing can pose as a barrier for screening in some patients. We hope our case will add to the growing literature on MUTYH mutations and give providers additional information to consider when treating patients with multiple BCCs.

## Conflicts of interest

None disclosed.

## References

[1] PDQ Cancer Genetics Editorial Board (2002). PDQ Cancer Information Summaries.

[2] Kilgour J.M., Jia J.L., Sarin K.Y. (2021). Review of the molecular genetics of basal cell carcinoma; inherited susceptibility, somatic mutations, and targeted therapeutics. Cancers.

[3] Spiker A.M., Troxell T., Ramsey M.L. (2025). StatPearls.

[4] Cho H.G., Kuo K.Y., Li S. (2018). Frequent basal cell cancer development is a clinical marker for inherited cancer susceptibility. JCI Insight.

[5] Bouso F., Zaher A. (2024). Genetic complexity in recurrent basal cell carcinoma: a MUTYH variant case report. Cureus.

[6] Vogt S., Jones N., Christian D. (2009). Expanded extracolonic tumor spectrum in MUTYH-associated polyposis. Gastroenterology.

[7] Tzoumpa S., Sevenet N., Bejar-Ardiles C. (2023). Multiple basal cell carcinomas revealing a biallelic MUTYH gene mutation in a 39-year-old male patient. J Eur Acad Dermatol Venereol.

[8] Syngal S., Brand R.E., Church J.M. (2015). ACG clinical guideline: genetic testing and management of hereditary gastrointestinal cancer syndromes. Am J Gastroenterol.

[9] Hodan R., Gupta S., Weiss J.M. (2024). Genetic/familial high-risk assessment: colorectal, endometrial, and gastric, version 3.2024, NCCN clinical practice guidelines in oncology. J Natl Compr Canc Netw.

[10] Richards S., Aziz N., Bale S. (2015). Standards and guidelines for the interpretation of sequence variants: a joint consensus recommendation of the American College of Medical Genetics and Genomics and the Association for Molecular Pathology. Genet Med.

